# Molecular Profiles of Antimalarial Drug Resistance in *Plasmodium* Species from Asymptomatic Malaria Carriers in Gia Lai Province, Vietnam

**DOI:** 10.3390/microorganisms13092101

**Published:** 2025-09-09

**Authors:** Hương Giang Lê, Tuấn Cường Võ, Jung-Mi Kang, Chau Van Khanh, Nguyen Thi Minh Trinh, Nguyen Thi Lien Hanh, Minkyoung Cho, Huynh Hong Quang, Byoung-Kuk Na

**Affiliations:** 1 Department of Parasitology and Tropical Medicine, Department of Convergence Medical Science, and Institute of Medical Science, Gyeongsang National University College of Medicine, Jinju 52727, Republic of Korea; gianglee291994@gmail.com (H.G.L.); vtcuong241@gmail.com (T.C.V.); jmkang@gnu.ac.kr (J.-M.K.); mcho@gnu.ac.kr (M.C.); 2 Tropical Diseases Clinical and Treatment Research Department, Institute of Malariology, Parasitology, and Entomology Quy Nhon, Quy Nhon 590000, Vietnam; cvkhanh01@gmail.com (C.V.K.); nguyenminhtrinh1983@gmail.com (N.T.M.T.); lienhanhnguyen84@gmail.com (N.T.L.H.)

**Keywords:** malaria, asymptomatic infection, *Plasmodium* species, Vietnam, antimalarial drug resistance

## Abstract

Asymptomatic malaria infection is a major concern in the fight against malaria, as it can act as a significant reservoir for its silent spread or transmission. Therefore, surveillance to detect asymptomatic subjects, particularly in regions with high malaria endemicity, is essential. This study aimed to investigate the status of asymptomatic submicroscopic malaria infections in Gia Lai province, Vietnam, and to analyze molecular profiles of antimalarial drug resistance in the parasites from the asymptomatic carriers. A total of 2171 individuals were included from three districts of Gia Lai province, Vietnam, an area where malaria is endemic. Asymptomatic submicroscopic infection was confirmed by quantitative real-time PCR, and the infected *Plasmodium* species were confirmed by sequencing. Antimalarial drug-resistant genes, including *pfk13*, *pfcrt*, *pvmdr-1*, and *pvcrt-o*, were analyzed in the parasites from asymptomatic cases. The rate of asymptomatic submicroscopic malaria infection was 2.67%. *P. falciparum* and *P. vivax* mono-infections, as well as mixed infections of *P. falciparum* and *P. vivax*, were identified, with *P. vivax* being more prevalent, a significant observation given the challenge of *P. vivax* relapses and its contribution to sustained malaria transmission. Adults, including young, middle-aged, and older adults, were the predominant affected groups. Asymptomatic infections were more common in females than in males. Interestingly, high frequencies of mutations in genetic markers associated with antimalarial drug resistance, particularly *pfk13* (C580Y, 100%), *pfcrt* (M74I/N75E/K76T, 100%), and *pvmdr-1* (F1076L, 100%), were observed in asymptomatic individuals, which may increase the risk of spreading drug resistance. These findings emphasize the urgent necessity for improved surveillance and targeted intervention to prevent the silent spread of malaria, supporting the National Malarial Control and Elimination Program in formulating malaria elimination strategies for Vietnam.

## 1. Introduction

Despite the decline in global malaria cases and deaths over the past decades, the disease continues to impose a significant public health burden on many countries in tropical and sub-tropical regions [1]. In 2023, global malaria incidences and deaths were estimated at 263 million cases and 597,000, respectively [2]. Countries belonging to the Greater Mekong Subregion (GMS), one of the malaria-endemic regions, aimed to eliminate malaria by 2030, with the specific target of eradicating *Plasmodium falciparum* by 2025, and have made significant progress in reducing malaria incidences and deaths in the recent two decades [3]. However, the spread of antimalarial drug resistance and the presence of asymptomatic carriers continue to pose significant threats to the effective control and elimination of malaria in the GMS [3,4,5].

Asymptomatic malaria carriers pose a significant challenge to malaria elimination programs as they serve as hidden transmission reservoirs [6,7]. Typically lacking visible malaria symptoms, asymptomatic carriers raise concerns for incorrect diagnosis and inappropriate treatment [7,8], and they contribute to ongoing transmission cycles within communities or regions, complicating efforts to interrupt malaria transmission and elimination [9]. Asymptomatic individuals harboring antimalarial drug-resistant parasites are also a greater concern as they can contribute to the ongoing transmission and spread of antimalarial drug resistance [10,11]. Therefore, understanding the prevalence and distribution of asymptomatic carriers in endemic areas is crucial for designing future intervention strategies for malaria elimination. The prevalence of asymptomatic carriers in malaria-endemic countries ranges from 0.4% to 90.6% [12]. A significant prevalence of asymptomatic carriers has also been reported in GMS countries [13,14,15,16], indicating that asymptomatic and/or submicroscopic malaria cases are common in these countries. Similar to other countries in the GMS, the Vietnamese Government launched the National Malaria Control and Elimination Program (NMCEP) to eliminate malaria by 2030 [17], and the malaria incidence rate in Vietnam has significantly declined over the past decade [18]. However, the Central Highlands remains a malaria hotspot, particularly Gia Lai province, which accounted for 66.2%, 62.3%, and 50.7% of total malaria cases in Vietnam over the successive years of 2020, 2021, and 2022. *P. falciparum* and *P. vivax* are the predominant malaria parasites circulating in the region [19]. Recently, an increased number of asymptomatic malaria cases have been documented in the Central Highlands of Vietnam [20,21,22], signifying that asymptomatic carriers are a major barrier to halting malaria transmission in the area and warrant more extensive research to comprehend the prevalence of asymptomatic infection there.

In this study, we investigated the prevalence and epidemiological characteristics of asymptomatic submicroscopic malaria infections in three districts of Gia Lai province, Vietnam, K’Bang, Ia Pa, and Krong Pa. Molecular analyses of antimalarial drug resistance genetic markers, including *P. falciparum* kelch-13 (*pfk13*) and chloroquine resistance transporter (*pfcrt*) and *P. vivax* multidrug resistance protein-1 (*pvmdr-1*) and orthologs of *pfcrt* (*pvcrt-o*), of malaria parasites detected in asymptomatic carriers were also performed. The findings indicate significant levels of asymptomatic carriers as well as high mutation rates of antimalarial drug resistance genetic markers among these carriers.

## 2. Materials and Methods

### 2.1. Study Area and Study Design

**Figure 1:**
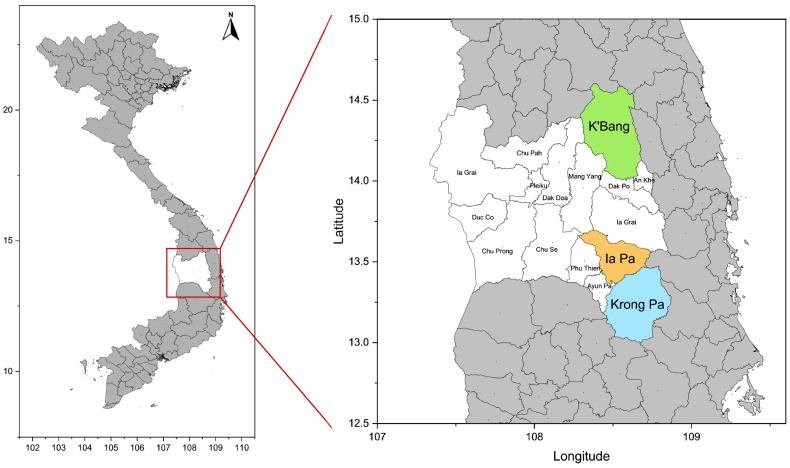
**Map of sample collection sites.** Three districts (K’Bang, Ia Pa, and Krong Pa) in Gia Lai province, Vietnam, are depicted in different colors. The map was created using Origin ver. 10.1 (OriginLab Corporation, Northampton, MA, USA).

This study was conducted in K’Bang, Ia Pa, and Krong Pa, located in Gia Lai Province, the Central Highlands of Vietnam (Figure 1). The province typically experiences an annual malaria season from June to October, peaking in September, with *P. falciparum* and *P. vivax* as the dominant species [23,24]. A cross-sectional survey was conducted in the 3 districts from November 2022 to April 2023. Participants enrolled in this study were randomly invited from healthy persons who visited the district health centers in each district, Gia Lai province, and they agreed to participate in this study. The questionnaire collecting demographic information, current clinical symptoms related to malaria, knowledge about malaria, i.e., the participant knew or heard about malaria, and past malaria infection history was administered to all participants (Table S1).

### 2.2. Sample Collection

Finger-prick capillary blood samples were collected from each individual for a rapid diagnostic test (RDT; SD Bioline Malaria Ag Pf/Pv, Standard Diagnostics, Seoul, Republic of Korea). All individuals tested negative in the RDTs. The results from microscopic examination using both thin and thick smears were also negative. Each blood sample was deposited onto 3 MM filter paper (Whatman, Buckinghamshire, UK) and air-dried for subsequent polymerase chain reaction (PCR) analysis. Each blood spot was individually stored in a zip-closure plastic bag with silica gel beads until required for use.

### 2.3. Detection of Asymptomatic Submicroscopic Infections

**Figure 2:**
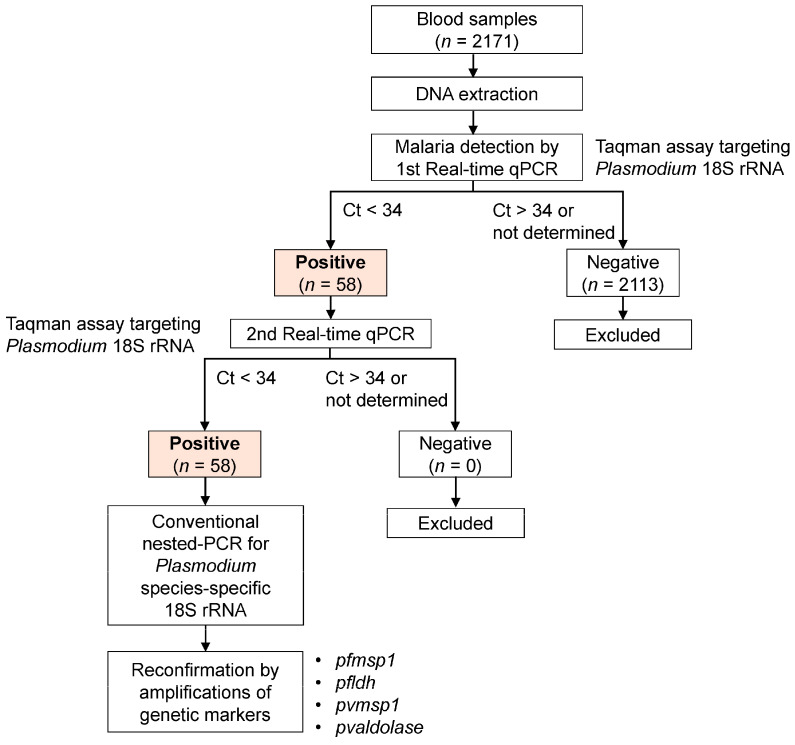
**Flowchart of study.** DNA extracted from blood spots collected from individuals was analyzed by two rounds of real-time PCRs to detect *Plasmodium* parasites. Malaria infection determined by real-time PCR analyses was presented as positive (infection) or negative (no infection). *Plasmodium* species were further identified using nested-PCR followed by sequencing of specific genetic markers. *n*, number of samples; Ct, cycle threshold.

A flowchart of asymptomatic carrier detection is summarized in Figure 2. Genomic DNA was extracted from each blood spot using the QIAamp DNA Blood Mini Kit (Qiagen, Hilden, Germany). Malaria detection employed quantitative real-time PCR (qPCR) targeting *Plasmodium* 18S ribosomal RNA (rRNA) with a previously described primer [25]. Probes were slightly modified: 5′-ATGGCCGTTTTTAGT-3′, labeled with 5′FAM (6-carboxyfluorecein) for reporting, and 3′MGB/NFQ (minor groove binder) as the quencher. The reaction was conducted in a 10 µL mixture containing 3 µL DNA, 600 nM of each primer, 200 nM probe, and 1× TaqMan^TM^ Universal PCR Master Mix (Applied Biosystems, Waltham, MA, USA) using the QuantStudio real-time PCR system (Applied Biosystems, Waltham, MA, USA). The thermal profile for qPCR was described previously [25]. The sensitivity of the qPCR assay was assessed using standard DNA from a *P. falciparum* 3D7 laboratory line (gifted by Professor Youn-Kyoung Goo, Kyungpook National University, Daegu, Korea). DNA was extracted from highly synchronized ring-stage parasites (ring stage > 95%, parasitemia: 6%), and 10-fold serial dilutions (up to 10^−10^ fold) were performed to create a standard curve (Figure S1). Based on this standard, detection was consistently achieved with a limit of detection (LOD) of 0.069 parasites/µL. The acceptance threshold for PCR performance was set 10 times higher than the LOD at 0.69 parasites/µL, equivalent to 3.45 parasites/µL per PCR reaction. All qPCR assays for each sample were performed in duplicate over two independent experiments, and samples with cycle threshold (Ct) values exceeding the acceptance threshold or indeterminate were categorized as negative for *Plasmodium* infection. A positive control DNA from diluted 3D7 was included in all assays. Species determination for *Plasmodium* in all qPCR-positive samples was conducted using conventional nested-PCR as previously described [26]. The amplified PCR products were analyzed on 2% agarose gel and visualized under ultraviolet light. Positive PCR products were verified by nucleotide sequencing analysis. *Plasmodium* infections were further confirmed by successful amplification of merozoite surface protein 1 (*msp1*) and lactate dehydrogenase (*ldh*) from *P. falciparum* and *msp1* and *aldolase* from *P. vivax* [27,28,29].

### 2.4. Molecular Analyses of Antimalarial Drug Resistance Genes

Antimalarial drug resistance genes, including the propeller domain of *pfk13*, 72–76 codons for *pfcrt,* and putative CQ resistance markers in *P. vivax* such as *pvmdr-1* and *P. vivax* orthologs of *pfcrt* (*pvcrt-o*), were analyzed from malaria-positive samples. These genes were amplified, sequenced, and analyzed as previously described [5].

### 2.5. Statistical Analysis

Independent demographic variables included age (classified into 5 groups), gender, ethnicity (Kinh, Ja Rai, Tay, Nung, Thai, and Bana), knowledge on malaria (i.e., infection route, typical symptoms, and prevention methods), and past malaria infection history. The dependent variable was malaria infection, determined by PCR analysis. Differences in demographic characteristics and malaria prevalence were assessed using Pearson’s chi-squared tests (χ^2^) with Bonferroni corrections through IBM SPSS ver.29.0. Multiple logistic regression analysis was conducted to explore factors such as age, gender, past malaria infection, and malaria knowledge in relation to asymptomatic cases using GraphPad Prism software version 10.2 (Boston, MA, USA). A significant threshold of *p* < 0.05 was established for all tests.

### 2.6. Ethics Approval

The study protocol was reviewed and approved by the Institutional Review Board of the Institute of Malariology, Parasitology, and Entomology, Quy Nhon, Vietnam (No. 637/VSR-NCDT). Verbal informed consent was obtained from all participants. For young children less than 15 years old, informed consent was obtained from their legal guardians. All methods were performed in accordance with the relevant guidelines and regulations.

## 3. Results

### 3.1. Demographic Characteristics of the Study Population

A total of 2171 individuals were enrolled: 438 from K’Bang, 310 from Ia Pa, and 1423 from Krong Pa (Table 1). Participants were categorized into five age groups: children (0–12 years), youths (13–25 years), young adults (26–44 years), middle-aged adults (45–60 years), and older adults (>60 years). The percentages of males and females were 57.58% (1250/2171) and 42.42% (921/2171), respectively. A majority (93.78% or 2036/2171) belonged to ethnic minority groups, with the Kinh people representing 6.22% (135/2171). None of the individuals showed clinical symptoms of malaria, such as fever, headache, fatigue, or myalgia, at the time of enrollment. Nearly all participants (94.33%, 2048/2171) had knowledge of malaria. A survey found that 20.87% (453/2171) of the participants had experienced a previous malaria infection, while the remaining 79.13% (1718/2171) had not.

### 3.2. Prevalence of Asymptomatic Submicroscopic Infections

All 2171 participants tested negative for malaria by both RDTs and microscopic tests at the time of blood collection. The prevalence of asymptomatic submicroscopic infections in Gia Lai, detected by qPCR, was 2.67% (58/2171) (Table 2). Asymptomatic cases were identified in Krong Pa and K’Bang, but not in Ia Pa. The mean parasite densities in asymptomatic cases from Krong Pa and K’Bang were 0.31 parasites/µL (range 0.11–1.05 parasites/µL) and 0.3 parasites/µL (range 0.11–2.27 parasites/µL), respectively. The infected *Plasmodium* species in 58 samples was further confirmed by specific gene amplifications (Figure S2). The proportions of *P. falciparum* and *P. vivax* mono-infections were 1.06% (23/2171) and 1.38% (30/2171), respectively. Co-infection with both *P. falciparum* and *P. vivax* was 0.23% (5/2171). No infection of *P. malariae*, *P. knowlesi*, or *P. ovale* was detected. The frequencies of asymptomatic infection varied significantly across districts. In Krong Pa, the prevalences of *P. falciparum*, *P. vivax*, and mixed infections of *P. falciparum*/*P. vivax* (*Pf*/*Pv*) were 0.49% (7/1432), 0.91% (13/1432), and 0.28% (4/1432), respectively. A higher proportion of *P. falciparum* and *P. vivax* infections was recorded in K’Bang at 3.65% (16/438) and 3.88% (17/438), respectively, while there was only one case of mixed infection of *Pf/Pv* (Table 2).

### 3.3. Analysis of Asymptomatic Infections by Demographic Characteristics

**Figure 3:**
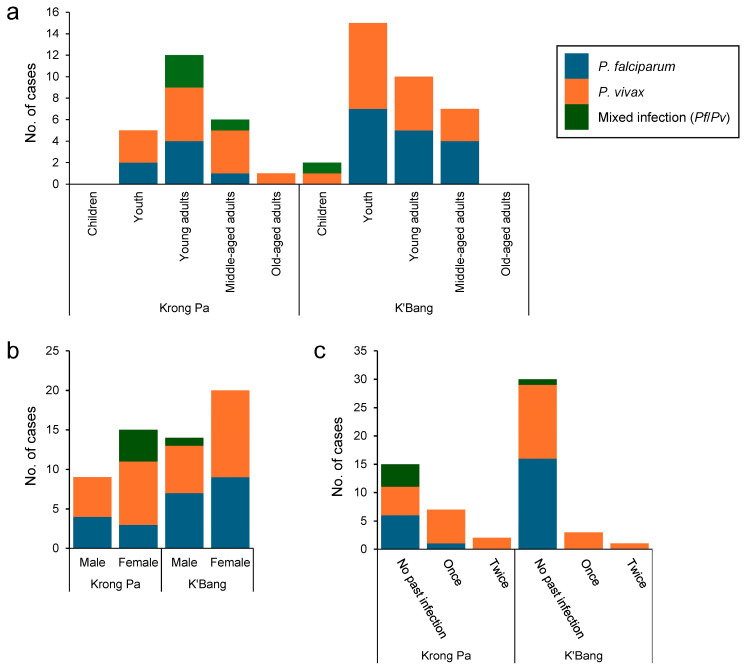
**Demographic analysis of asymptomatic submicroscopic cases.** (**a**) Distribution of asymptomatic cases by district and age. (**b**) Ratio of males to females. (**c**) History of past malaria infections.

Asymptomatic carriers were predominantly observed in adults, exhibiting distinct patterns across different age groups and genders (Table 3). Young adults and youths, with prevalences of 1.01% (OR: 1.07, 95% CI: 0.58 to 1.99, *p* = 0.83) and 0.92% (OR: 0.03, 95% CI: 0.02 to 0.05, *p* < 0.001), respectively, were the most affected groups, followed by middle-aged adults at 0.60% (OR: 1.24, 95% CI: 0.59 to 2.5, *p* = 0.55). All adult subsets in Krong Pa displayed elevated rates of asymptomatic *P. vivax* infections, whereas old-aged adults did not show asymptomatic *P. falciparum* infections (Figure 3a). Asymptomatic mixed infections with *P. falciparum* and *P. vivax* occurred solely in young and middle-aged adults. In K’Bang, all age groups except the old-aged displayed asymptomatic submicroscopic infections, predominantly among youths and young adults. A mixed infection case emerged in children. Female individuals in both Krong Pa and K’Bang demonstrated higher asymptomatic infection rates than males, at 1.61% (OR: 2.11, 95% CI: 1.24 to 3.64, *p* = 0.006) and 1.06% (OR: 0.02, 95% CI: 0.01 to 0.03, *p* < 0.001), respectively (Figure 3b, Table 3). Most asymptomatic cases occurred in individuals without prior exposure to malaria but were not statistically significant (OR: 1.13, 95% CI: 0.61 to 2.09, *p* = 0.74) (Figure 3c, Table 3). However, most individuals with a history of malaria exhibited asymptomatic *P. vivax* mono-infections (40%) (Figure 3c).

### 3.4. Molecular Analyses of Antimalarial Drug Resistance Markers in P. falciparum and P. vivax from Asymptomatic Carriers

**Figure 4:**
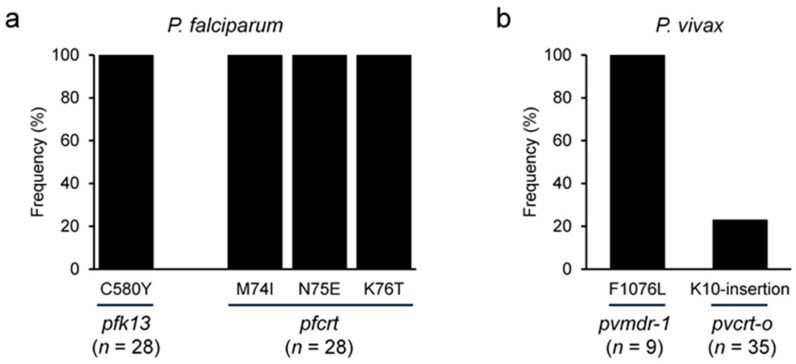
**Antimalarial drug resistance profiles.** Antimalarial drug resistance genes were amplified and analyzed through sequencing. Major mutations associated with antimalarial drug resistance were identified in *pfk13* and *pfcrt* in *P. falciparum* (**a**) and *pvmdr-1* and *pvcrt-o* in *P. vivax* (**b**) from asymptomatic carriers. Note that *pvmdr-1* is the result of only 9 successfully amplified samples among 35 samples.

The *pfk13*, *pfcrt*, and *pvcrt-o* were successfully amplified from all asymptomatic infection samples. These amplified products were cloned and sequenced to assess the parasites’ antimalarial drug resistance. For *P. falciparum* (*n* = 28), the C580Y mutation in *pfk13*, mainly associated with artemisinin resistance, and the M74I/N75E/K76T mutations in *pfcrt,* indicative of chloroquine (CQ) resistance, were observed in all samples (100%, 28/28) (Figure 4a). In *P. vivax* (*n* = 35), the K10 insertion in *pvcrt-o* was found in eight samples, constituting 22.9% of the cases. In contrast, *pvmdr-1* was successfully amplified and sequenced in only nine samples, all of which harbored the F1076L mutation (100%, 9/9) (Figure 4b).

## 4. Discussion

This study highlighted a significant proportion of asymptomatic submicroscopic individuals in Gia Lai province, Vietnam. The overall prevalence of asymptomatic submicroscopic infections in Gia Lai province was 2.67% (58 positive cases out of 2171). However, this varied by district: K’Bang (7.76%, 34 positive cases out of 438), Krong Pa (1.68%, 24 positive cases out of 1432), and Ia Pa (0.00%, 0 positive cases out of 310). The uneven number of participants across these districts may be a limitation of this study, but given the varied populations and the distinct accessibility of sampling in each district, the results provide valuable insights into the spread of asymptomatic infections in Gia Lai province. A higher prevalence of asymptomatic cases (4.4%) was recently reported in three districts of Dak Nong province, Vietnam: Dak Buk So (1.7%, 22/1328), Dak Ngo (3.5%, 31/890), and Quang Truc (12.2%, 72/591) [22]. Meanwhile, the prevalence of asymptomatic cases detected in this study exceeded that reported from Gia Lai province in 2016−2017 (1.741%) [21]. A considerable prevalence of submicroscopic malaria patients was also observed in Nam Tra My district, Quang Nam province, Central Vietnam [20]. Collectively, these results suggest that asymptomatic submicroscopic malaria cases are commonly present in the Central Highlands, Vietnam, despite challenges in attaining an accurate prevalence figure in each province due to variations in the methods used to detect asymptomatic cases. This assertion is further supported by earlier surveys on submicroscopic malaria infections in South-Central Vietnam [20,30,31,32]. Therefore, the use of molecular assays such as PCR to improve the diagnostic sensitivity for the submicroscopic infections is highly recommended.

It has been demonstrated that both symptomatic and asymptomatic cases are generally higher in males than in females, attributed to their greater involvement in outdoor working activities, which increases exposure to infection [13,15,33,34,35]. However, this study revealed that asymptomatic cases were more common among females than males in both Krong Pa and K’Bang districts. Asymptomatic malaria infection in pregnant women could cause a higher risk of anemia, stillbirth, miscarriage, and preterm delivery [9,36]. It remains unclear why females exhibit a higher prevalence of asymptomatic cases than males in the studied area. However, the significance of asymptomatic infection in females, particularly in pregnant women [9,37,38], warrants further investigation. Active preventive measures, such as using long-lasting insecticide-treated nets (LLINs) and indoor residual spray, have proven highly effective in reducing the prevalence of malaria infections and asymptomatic cases, especially in rural settings [39]. The Vietnamese Government also recommends the use of LLINs in Gia Lai to prevent mosquito bites, but the actual usage rate of LLINs in the study population has not been confirmed. In Dak Nong province, however, it has been reported that only a low percentage of residences (41.3% to 59.9%) used LLINs daily despite the majority (up to 95%) possessing LLINs [22]. A similar situation is expected in Gia Lai province. Therefore, strong administrative support and an education program should be implemented to encourage the use of LLINs and/or other preventive measures. Interestingly, most asymptomatic cases detected in both Krong Pa and K’Bang districts were reported by individuals with no past malaria infections: Krong Pa (62.5%, 15/24) and K’Bang (88.2%, 30/34). Mono-infections of either *P. vivax* or *P. falciparum*, and mixed infections of *Pf*/*Pv*, were identified in the population. Meanwhile, *P. vivax* was the common species in asymptomatic cases who reported having had past malaria infections. No significant difference was found in parasite density between the two groups: no past infection (0.11−1.05 parasites/μL) and past infection (0.12−2.27 parasites/μL). It remains unclear why asymptomatic cases were more common in the population with no past malaria infection. A plausible explanation is that individuals with prior malaria exposure might have acquired partial immunity that either prevents asymptomatic carriage or leads to symptomatic episodes requiring treatment, thereby decreasing the probability of detecting asymptomatic parasitemia [40], but further investigation is necessary.

Asymptomatic infections of *P. vivax* (1.38%) were more frequent than those of *P. falciparum* (1.06%) in Gia Lai province. Similar phenomena were also observed in GMS countries, including Cambodia, Myanmar, and Thailand [15,33,41,42]. The dominance of *P. vivax* in asymptomatic individuals may stem from its ability to form dormant hypnozoites that can reactivate, causing relapses and contributing to the persistent spread of the infection [43]. Moreover, *P. vivax* parasites tend to infect reticulocytes, which are fewer in number compared to mature red blood cells targeted by *P. falciparum*. This results in lower parasite densities and either no or milder clinical manifestations [44]. To address asymptomatic *P. vivax* infections, intensified interventions such as proper drug administration, enhanced diagnostic tools capable of detecting low parasite densities, and robust surveillance systems are essential.

Antimalarial drug-resistant parasites in asymptomatic cases could lead to serious and detrimental consequences for the effective control and elimination of malaria through silent transmission. Antimalarial drug resistance among asymptomatic individuals has been reported in Southeast Asia [21,45] and Sub-Saharan African countries [10,46,47]. Notably, artemisinin resistance has resulted in a significant increase in asymptomatic *P. falciparum* infections in the GMS. These antimalarial drug-resistant parasites can remain in the bloodstream at low levels, often without symptoms due to partial immunity [48,49]. This study revealed that *P. falciparum* in all asymptomatic individuals carried C580Y in *pfk13* and a triple mutant of M74I/N75E/K76T in *pfcrt*, indicating potential artemisinin and CQ resistance. A high frequency of F1076L in *pvmdr-1* and a substantial frequency of K10-insertion in *pvcrt-o* were also identified in *P. vivax* from asymptomatic cases. The *P. vivax* drug resistance in Asia, including Vietnam, was broadly reported [50,51]. The genetic profiles of antimalarial drug-resistant markers of *P. vivax* in Asian countries, including Vietnam, have been investigated [5,38,50,51,52]. Given that CQ remains the first-line drug for treating vivax malaria in Vietnam, these mutations in *P. vivax* in asymptomatic cases are alarming. These findings suggest that asymptomatic individuals may act as silent reservoirs for antimalarial drug-resistant parasites, facilitating the spread and transmission of antimalarial drug resistance in Gia Lai province. Expanded surveillance of asymptomatic cases followed by appropriate treatment is necessary.

In conclusion, this study highlights a substantial level of asymptomatic submicroscopic malaria infections in Gia Lai province, Vietnam, and a high frequency of antimalarial drug resistance, including resistance to artemisinin, among these asymptomatic individuals. It is a limitation of this study that only three districts in Gia Lai province were included. Considering Gia Lai province is the most important malaria hotspot in Vietnam, further research in broader areas in Gia Lai province is warranted to gain an in-depth understanding of the overall prevalence of asymptomatic submicroscopic infections and their contribution to silent malaria transmission in the province. The high frequency of mutations associated with antimalarial drug resistance in asymptomatic infections also emphasized the necessity to investigate the overall antimalarial drug resistance status in the area. The findings of this study will assist the NMCEP in designing and planning effective malaria elimination strategies for Vietnam. It is recommended to investigate asymptomatic reservoirs through larger community-based surveys in malaria-endemic regions of Vietnam to accurately estimate the burden of malaria and optimize control interventions of the disease. Application of molecular methods to enhance diagnostic sensitivity is also necessary to detect submicroscopic infections.

## Figures and Tables

**Table 1:** Demographical characteristics of the study population.

Categories	Krong Pa (*n* = 1423)		Ia Pa (*n* = 310)		K’Bang (*n* = 438)		Gia Lai (*n* = 2171)		*p*-Value
	*n*	%	*n*	%	*n*	%	*n*	%	
**Age (in years)**									
Children (0–12)	42	2.95	55	17.74	9	2.05	106	4.88	<0.001
Youth (13–25)	536	37.67	67	21.61	163	37.21	766	35.28	
Young adults (26–44)	522	36.68	82	26.45	184	42.01	788	36.30	
Middle-aged adults (45–60)	259	18.20	67	21.61	77	17.58	403	18.56	
Old-aged adults (>60)	64	4.50	39	12.58	5	1.14	108	4.97	
**Gender**									
Male	907	63.74	147	47.42	196	44.75	1250	57.58	<0.001
Female	516	36.26	163	52.58	242	55.25	921	42.42	
**Ethics**									
Ja Rai	1356	95.29	277	89.35	0	0.00	1633	75.22	<0.001
Kinh	67	4.71	31	10.00	37	8.45	135	6.22	
Tay	0	0.00	1	0.32	3	0.68	4	0.18	
Nung	0	0.00	0	0.00	2	0.46	2	0.09	
Thai	0	0.00	0	0.00	3	0.68	3	0.14	
Bana	0	0.00	1	0.32	393	89.73	394	18.15	
**Knowledge on malaria**									
Yes	1352	95.01	265	85.48	431	98.40	2048	94.33	<0.001
No	71	4.99	45	14.52	7	1.60	123	5.67	
**Past malaria infections**									
0	1064	74.77	289	93.23	365	83.33	1718	79.13	<0.001
1	267	18.76	17	5.48	59	13.47	343	15.80	
2	89	6.25	4	1.29	12	2.74	105	4.84	
3	3	0.21	0	0.00	2	0.46	5	0.23	

Differences in demographic characteristics were determined using Pearson’s chi-squared tests with Bonferroni corrections by IBM SPSS 29.0.

**Table 2:** Prevalence of *Plasmodium* species detected in the three districts of Gia Lai province, Vietnam.

Species	Krong Pa (*n* = 1432)		K’Bang (*n* = 438)		Gia Lai (*n* = 2171)		*p*-Value
	*n*	%	*n*	%	*n*	%	
*P. falciparum*	7	0.49	16	3.65	23	1.06	<0.001
*P. vivax*	13	0.91	17	3.88	30	1.38	
Mixed infection (*Pf*/*Pv*)	4	0.28	1	0.23	5	0.23	
Total	24	1.68	34	7.76	58	2.67	

Differences in demographic characteristics were determined using Pearson’s chi-squared tests with Bonferroni corrections by IBM SPSS 29.0.

**Table 3:** Prevalence of malaria infection based on population categories in Gia Lai province.

Categories	Negative PCR		Positive PCR		Odds Ratio	95% CI	*p*-Value
	*n*	%	*n*	%			
**Age**							
Children (0–13)	104	4.79	2	0.09	0.72	0.11 to 2.51	0.66
Youths (13–25)	746	34.36	20	0.92	0.03	0.02 to 0.05	<0.001
Young adults (26–44)	766	35.28	22	1.01	1.07	0.58 to 1.99	0.83
Middle-aged adults (45–60)	390	17.96	13	0.60	1.24	0.59 to 2.50	0.55
Old-aged adults (>60)	107	4.93	1	0.05	0.35	0.02 to 1.69	0.31
**Gender**							
Male	1227	56.52	23	1.06	0.02	0.01 to 0.03	<0.001
Female	886	40.81	35	1.61	2.11	1.24 to 3.64	0.006
**Past infection**							
Yes	440	20.27	13	0.60	0.89	0.48 to 1.64	0.74
No	1673	77.06	45	2.07	1.13	0.61 to 2.09	
**Knowledge on malaria**							
Yes	1992	91.76	56	2.58	0.73	0.2 to 2.61	0.77
No	121	5.57	2	0.09	1.38	0.38 to 4.96	

Multiple logistic regression analysis was used to explore demographic characteristics in relation to asymptomatic infection using GraphPad Prism ver. 10.2. CI, confidence interval.

## Data Availability

The data supporting the conclusions of this article are provided within the article. The original datasets analyzed in this study are available from the corresponding author upon request. All data generated or analyzed during this study are included in this published article (and its Supplementary Information files). The sequence data obtained in this study are openly available in GenBank of NCBI at https://www.ncbi.nlm.nih.gov/ under the Accession No. PQ817457–PQ817545.

## Supplementary Materials

The following supporting information can be downloaded at https://www.mdpi.com/article/10.3390/microorganisms13092101/s1: Table S1: Questionnaire for epidemiological surveillance of asymptomatic malaria infections in Gia Lai, Vietnam; Figure S1: Standard curve analysis; and Figure S2: Amplifications of *P. falciparum* and *P. vivax* genes by nested-PCR.

## Abbreviations

The following abbreviations are used in this manuscript:

CI	confidence interval
CQ	chloroquine
Ct	cycle threshold
GMS	Greater Mekong Subregion
*msp-1*	*merozoite surface protein-1*
*ldh*	*lactate dehydrogenase*
LLINs	long-lasting insecticide-treated nets
LOD	limit of detection
NMCEP	National Malaria Control and Elimination Program
ORs	odd ratios
PCR	polymerase chain reaction
*pfcrt*	*P. falciparum chloroquine resistance transporter*
*pfk13*	*P. falciparum kelch-13*
*pvcrt-o*	*P. vivax chloroquine resistance transporter orthologs*
*pvmdr-1*	*P. vivax multidrug resistance protein-1*
RDT	rapid diagnostic test
qPCR	quantitative real-time PCR
rRNA	18s ribosomal RNA
