# Supplementary material for: Molecular Profiles of Antimalarial Drug Resistance in Plasmodium Species from Asymptomatic Malaria Carriers in Gia Lai Province, Vietnam
Source: Microorganisms. 2025 Sep 9;13(9):2101. doi: 10.3390/microorganisms13092101 (PMC12473079; doi:10.3390/microorganisms13092101)
Supplement: Supplementary file 1 [file microorganisms-13-02101-s001.zip › microorganisms-3848815-supplementary.pdf]

Questionnaire for epidemiological surveillance study of asymptomatic malaria infections in Gia Lai Province, Vietnam

<table>
<tr><th colspan="3">Participation agreement</th></tr>
<tr><td colspan="3">This study aims to surveil the epidemiological status of asymptomatic malaria infections among the residents in Gia Lai Province, Vietnam. If you agree to participate in this study, we will inform you more detail about the aim and process of this study. If you agree to participate in this study, you need to complete below questionnaire and donate a few drops of finger-prick blood samples for this study. Your personal information would be legally protected from release by the regulation. Do you agree to participate in this study?
☐ Yes, I agree.</td></tr>
<tr><th colspan="3">Location</th></tr>
<tr><td colspan="3">Province: Gia Lai
District: Krong Pa (KP) ☐ K'Bang (KB) ☐ Ia Pa (IP) ☐
Commune:</td></tr>
<tr><th colspan="3">Demographical information</th></tr>
<tr><td>Name:
Ethnic:
Study code:
Date:</td><td>Age:
Gender: Male ☐
Female ☐</td><td>Note:</td></tr>
<tr><th colspan="3">Travel history and knowledge about malaria</th></tr>
<tr><td>Did you live in or visit to other areas except Gia Lai in last one month?</td><td>Yes ☐</td><td>No ☐</td></tr>
<tr><td>Do you know about malaria? (infection route, typical symptoms, and prevention methods etc.)</td><td>Yes ☐</td><td>No ☐</td></tr>
<tr><td>Have you been infected with malaria before?</td><td>Yes ☐
How many times?</td><td>No ☐</td></tr>
</table>

<table>
<tr><th colspan="4">Health check: Do you have any symptoms below or any inconveniences?</th></tr>
<tr><td>Symptoms</td><td>Yes</td><td>No</td><td>If yes, detail</td></tr>
<tr><td>Fever</td><td>☐</td><td>☐</td><td></td></tr>
<tr><td>Chills</td><td>☐</td><td>☐</td><td></td></tr>
<tr><td>Sweat</td><td>☐</td><td>☐</td><td></td></tr>
<tr><td>Headache</td><td>☐</td><td>☐</td><td></td></tr>
<tr><td>Dizziness</td><td>☐</td><td>☐</td><td></td></tr>
<tr><td>Nausea or vomiting</td><td>☐</td><td>☐</td><td></td></tr>
<tr><td>Tiredness or fatigue</td><td>☐</td><td>☐</td><td></td></tr>
<tr><td>Muscle pain</td><td>☐</td><td>☐</td><td></td></tr>
<tr><td>Others</td><td>☐</td><td>☐</td><td></td></tr>
</table>

※ **If you have more than 1 among the above symptoms, you could be excluded in this study.**

For research staff only

Participant Code	
**Body temperature (°C)**	
**RDT**	*P. falciparum* ☐<br>*P. vivax* ☐<br>All negative ☐
**Microscopic examination (thick/thin)**	*P. falciparum* ☐<br>*P. vivax* ☐<br>Others ☐<br>Negative ☐
**Parasitemia (parasites/µl)**	*P. falciparum*:<br>*P. vivax*:<br>Others:
**Blood filter Code number**	

Supplement File 1: Table S1. Questionnaire for epidemiological surveillance of asymptomatic malaria infections in Gia Lai, Vietnam.

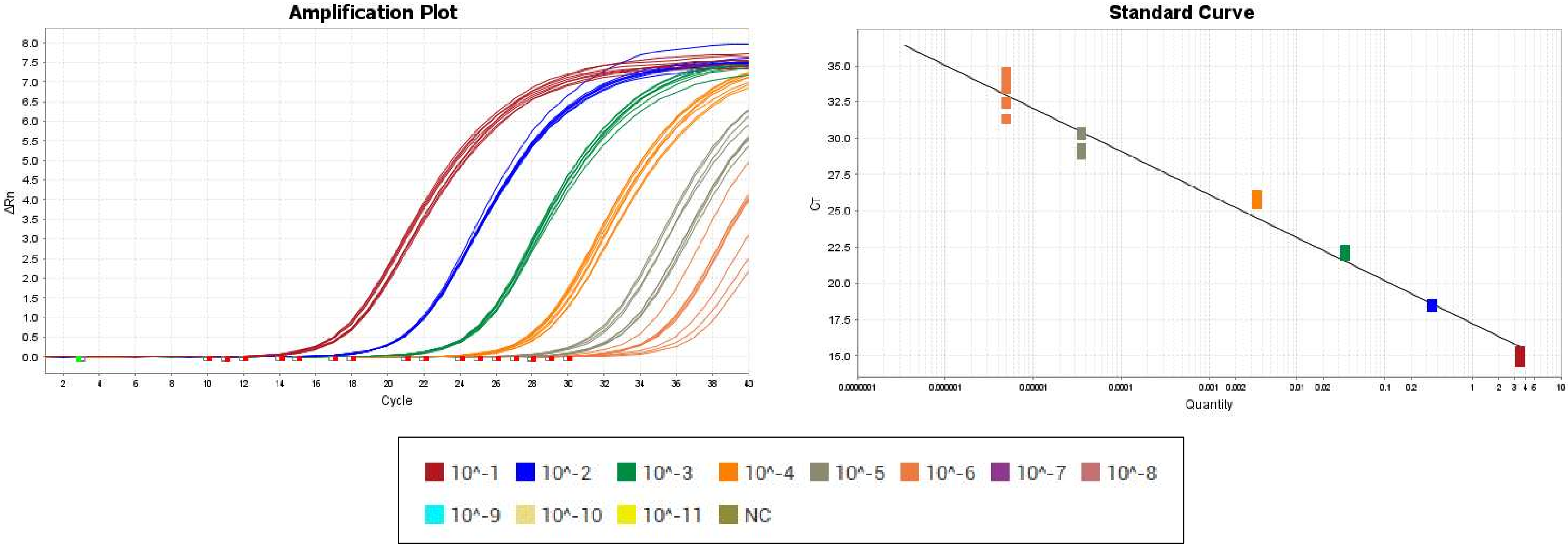

Supplement File 2: Fig. S1. Standard curve analysis. The amplification of 18S rRNA from *P. falciparum* 3D7 genomic DNA with 10-fold serial dilutions (up to 10^{-10} fold) was measured. The Ct value of each dilution was taken during 10 replications (left panel) and used to create the standard curve (right panel).

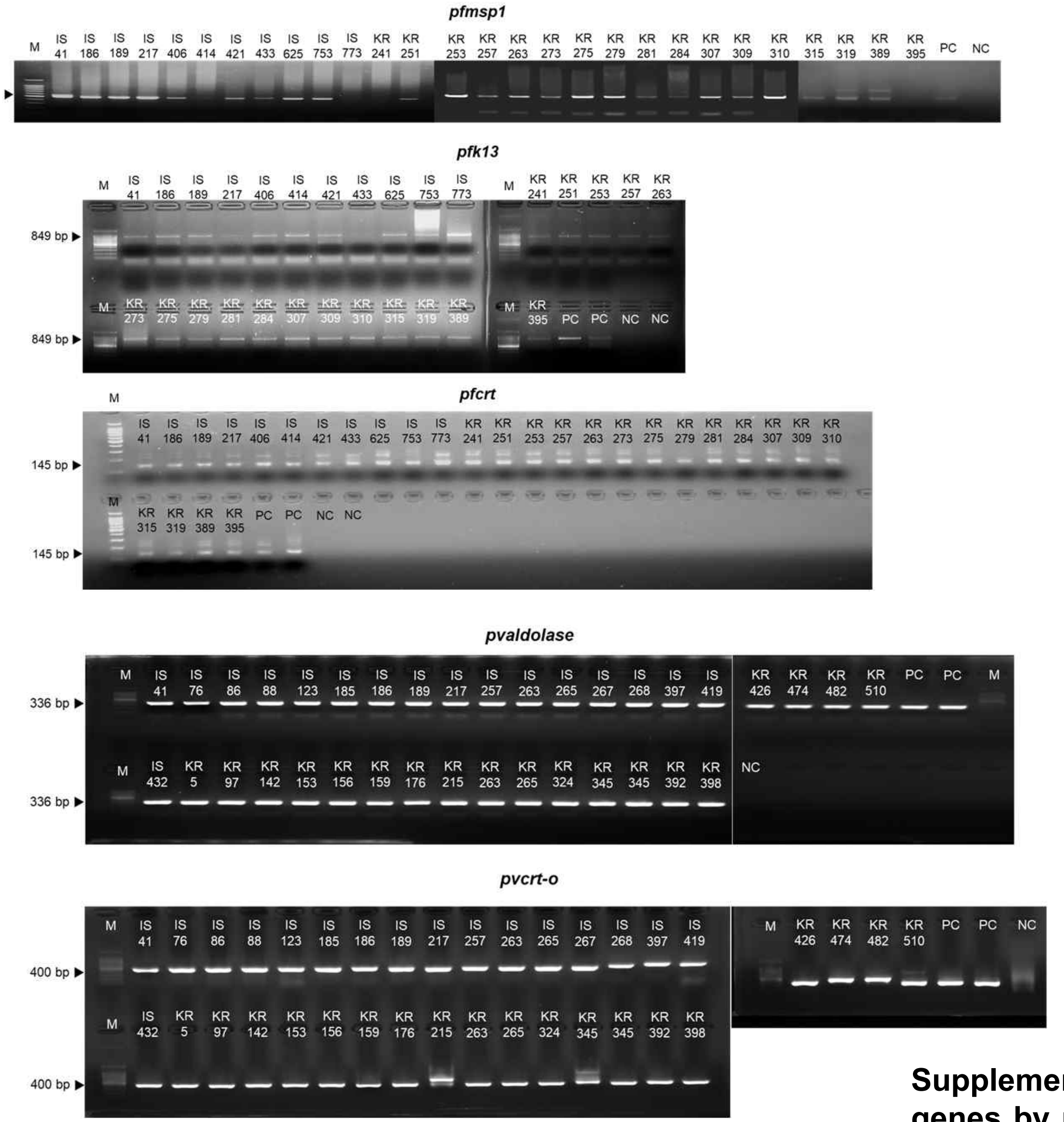

Supplement File 3: Fig. S2. Amplifications of *P. falciparum* and *P. vivax* genes by nested-PCR. PC, positive control; NC, Negative control; M, DNA marker.
